# Electricity

**Published:** 1858-10

**Authors:** 


### ELECTRICITY.

A correspondent asks our views of this subtle, mysterious, invisible agent. The articles in this Journal are written from our own personal experience and observation; we, therefore, have merely to say, that the only electricity we have any knowledge or experience of, of late years, is the electricity of a—kiss; when our little Alice, just four years old at this writing, comes softly into our study, and throwing her little dimpled arm around our neck, exclaims, with the kiss:

"Darling little fadder, I do love you so dearly, give me a piece o' candy!" If anybody can give us a better specimen of human nature broke loose, we will be glad to record it.

